# A Rare Type of Pneumothorax Recurrence After Surgery, Caused by a Lung Laceration Next to the Adhesion

**DOI:** 10.7759/cureus.72585

**Published:** 2024-10-28

**Authors:** Sachie Koike, Takayuki Shiina, Keiichirou Takasuna

**Affiliations:** 1 Thoracic Surgery, Ina Central Hospital, Ina, JPN

**Keywords:** adhesion, athlete, lung laceration, pneumothorax, recurrence

## Abstract

Recurrence of primary spontaneous pneumothorax after surgical treatment was often caused by overlooking bullae in surgical treatments, bullae regrowth, or bullae neogenesis. Herein, we present a very rare type of recurrence after surgical treatment, which was caused by lung laceration next to the adhesion created after the surgery. The patient was a 22-year-old volleyball player, and we presumed that sudden chest wall compression that occurred during volleyball displaced the lung next to the adhesion inwardly and caused the lung laceration.

## Introduction

Primary spontaneous pneumothorax (PSP) has been mainly treated with surgery or pleurodesis in cases of unsuccessful or recurrent conventional drainage, and bullectomy, cautery ablation with soft coagulation, suture plication, and loop ligation are performed as surgical treatment [1-4]. The risk of recurrence after surgical treatment has been reported, and the commonly reported causes include overlooking bullae in surgical treatments, bullae regrowth, or bullae neogenesis [5-9]. We present a very rare type of PSP recurrence after surgical treatment caused by lung parenchymal laceration next to lung adhesion.

## Case presentation

**Figure 1:**
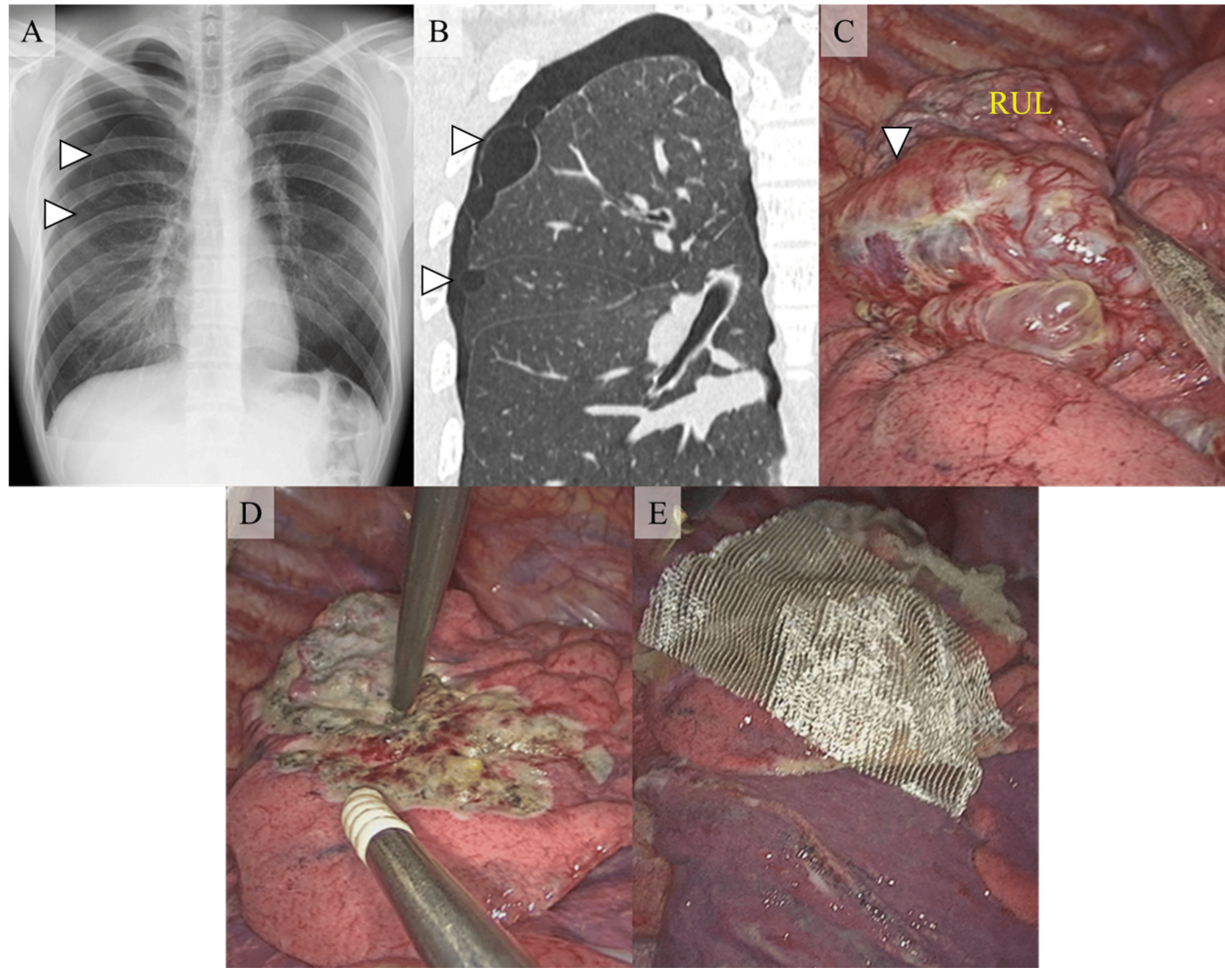
Radiological images and intraoperative findings of first surgical treatment A: Chest X-ray revealed right-side spontaneous pneumothorax; B: Chest computed tomography showed 7cm bulla in the right upper lobe and 1cm bulla in the right middle lobe; C: A large bulla arises from the cranial to the caudal part of the right upper lobe; D: Cautery ablation with soft coagulation of each bulla was performed; E: Almost the whole area of the right upper lobe is covered with ORC mesh sheets. ORC: Oxidized regenerated cellulose; RUL: Right upper lobe

A 22-year-old man was admitted to our hospital with chest pain, which continued for two days. He had a history of left spontaneous pneumothorax treated with bullectomy. Chest X-ray revealed right-side spontaneous pneumothorax (Figure 1A). Chest computed tomography showed a bulla with a diameter of 7cm in the right upper lobe and a bulla with a diameter of 1cm in the right middle lobe (Figure 1B). Chest tube insertion was done, and we found that the air leak had stopped. He had selected preventive surgical treatment to reduce the risk of the recurrence of pneumothorax. The video-assisted thoracoscopic surgery was performed. During the surgery, we found a large bulla that arises from the cranial to the caudal part of the right upper lobe (Figure 1C). Also, we found a small bulla in the right middle lobe. We performed cautery ablation with soft coagulation of each bulla. We also performed pleural covering of almost the whole area of the upper lobe and ablated area of the middle lobe with oxidized regenerated cellulose (ORC) mesh sheets (Figure 1D, 1E). The patient was discharged with complete resorption of the pneumothorax.

**Figure 2:**
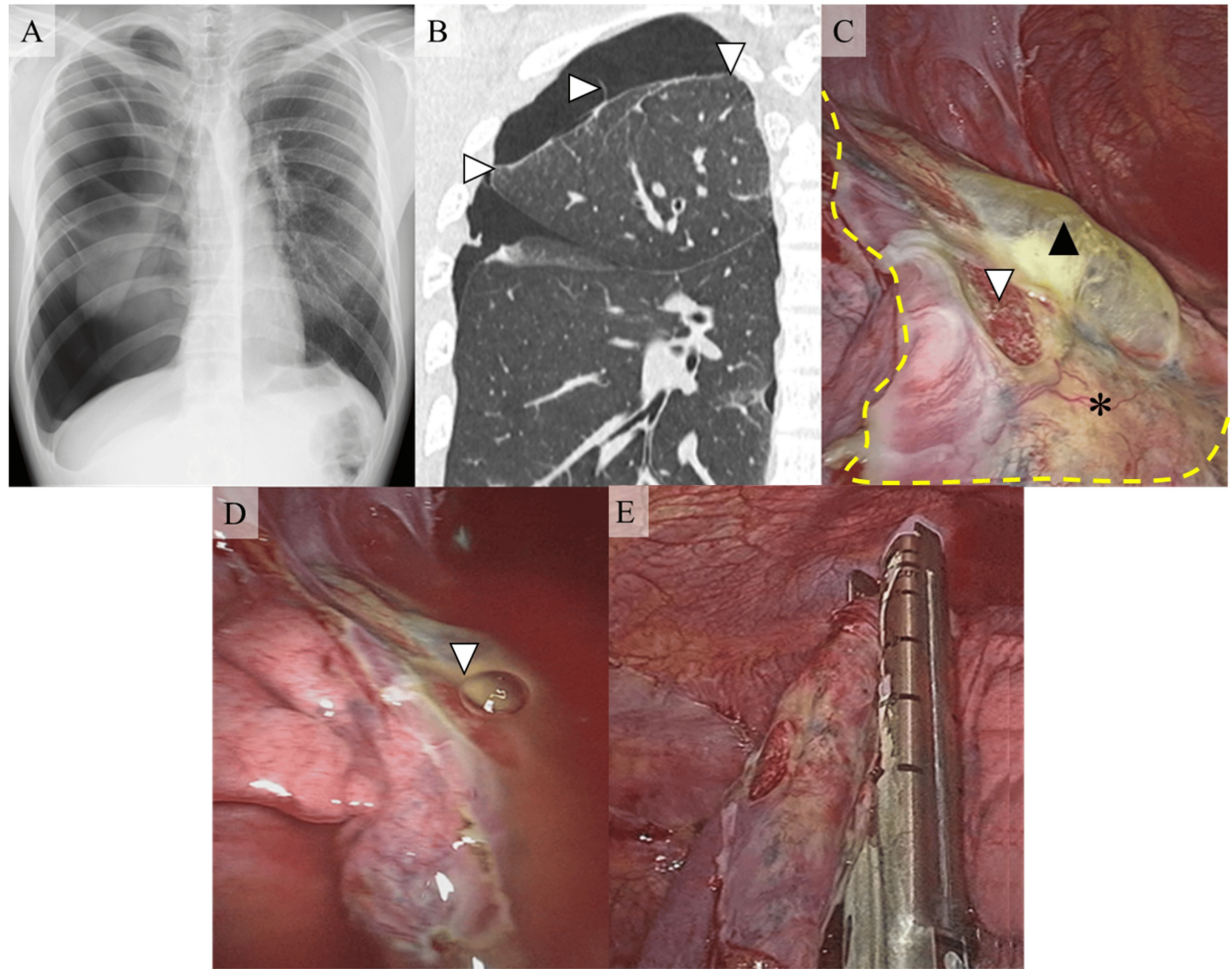
Radiological images and intraoperative findings of the second surgical treatment A: Chest X-ray revealed complete right-side spontaneous pneumothorax; B: Adhesion between the right upper lobe and chest wall (arrow) was found on chest computed tomography, and no obvious overlooked or regrown bullae were found; C: Lung laceration (white arrow) was found in the area without adhesion (*) next to the released adhesion (yellow line). A part of an ablated bulla or degenerated ORC mesh sheet was found nearby (black arrow); D: The air from the laceration (arrow) was found in the water-sealing test; E: The laceration was resected with an endo stapler. ORC: Oxidized regenerated cellulose

Two months later, he was admitted again to our department with severe chest pain, which continued for four days. Chest X-ray revealed complete right-side spontaneous pneumothorax (Figure 2A). Chest tube insertion was done, and air leakage was found. Chest computed tomography was performed after the lung had expanded. The image indicated the adhesion between the right upper lobe and chest wall, and no obvious overlooked or regrown bullae was found (Figure 2B). The air leak continued for a week, and the surgery was conducted. During the surgery, we found the adhesion between almost the whole surface of the right upper lobe and the chest wall. The adhesion of the lateral and dorsal parts of the upper lobe was released. We found the area without the adhesion (Figure 2C: area inside the yellow line), which consisted of a part of ablated bulla or degenerated ORC sheet (Figure 2C: black arrow) and normal lung parenchyma around it (Figure 2C: *). In the normal lung parenchyma area, we found a lung laceration (Figure 2C: white arrow) next to the released adhesion (Figure 2C: yellow line), and the sealing test revealed an air leak from the laceration (Figure 2D: arrow). We considered that the laceration was the cause of the PSP recurrence and resected the area with an endoscopic stapler (Figure 2E). The lung resected area was covered with ORC mesh sheets. The patient was discharged with complete resorption of the pneumothorax. Pathological examination of the resected lung revealed that only the lung laceration was in the specimen, and no ruptured bullae were found.

## Discussion

Surgical treatment for PSP has been performed in cases of unsuccessful or recurrent conventional drainage. Also, preventive surgical interventions have been performed in cases with bullae to reduce the risk of recurrence [1-4]. Bullectomy, cautery ablation with soft coagulation, suture plication, and loop ligation are performed as surgical treatment, and usually, pleural covering with sheet-type materials is implemented to reduce the recurrence [1-4,10]. The recurrence rate after surgical treatment was reported to be 2-14%, and the commonly reported causes are overlooking of bullae in surgical treatments, bullae regrowth, or bullae neogenesis [1-9].

Unlike previous reports, the cause of recurrence after surgery in the present case was the lung laceration created in the lung parenchyma without adhesion next to the adhesive area. No previous reports have presented the clinical condition like our case; this is the first report of such cases. We presumed that the patient's occupation, a volleyball player, might be one of the causes of this lung laceration. Wagner RB et al. classified lung laceration into four types and defined Type IV laceration as laceration caused by sudden chest wall compression displacing the lung inwardly next to thick pleuropulmonary adhesion [11,12]. During volleyball, the chest wall of a player is compressed when the ball hits the body or the player does a pancake. Smith LJ et al. suggested that the physical response to volleyball injuries may be similar to high-velocity seat belt injuries [13]. We hypothesized that the patient's chest wall was compressed during practice or a game of volleyball, the lung parenchyma without adhesion displaced inwardly, and the laceration was made due to the adhesion nearby.

## Conclusions

In conclusion, we present a very rare type of PSP recurrence after surgical treatment caused by lung parenchymal laceration next to lung adhesion. The cause of the lung laceration may be the adhesion formed after surgery and the chest compression that occurred during volleyball. The distinctiveness of the patient's occupation might cause the rare clinical condition. We should take care of lung lacerations when we perform PSP treatment related to lung adhesion (e.g., surgical treatment, pleurodesis) on athletes.
